# Transcriptional Response of Ovine Lung to Infection with Jaagsiekte Sheep Retrovirus

**DOI:** 10.1128/JVI.00876-19

**Published:** 2019-10-15

**Authors:** Anna Eleonora Karagianni, Deepali Vasoya, Jeanie Finlayson, Henny M. Martineau, Ann R. Wood, Chris Cousens, Mark P. Dagleish, Mick Watson, David J. Griffiths

**Affiliations:** a Moredun Research Institute, Edinburgh, United Kingdom; b Roslin Institute and Royal (Dick) School of Veterinary Studies, University of Edinburgh, Edinburgh, United Kingdom; Ulm University Medical Center

**Keywords:** JSRV, jaagsiekte, RNA-Seq, lung adenocarcinoma, sheep, cancer, ovine pulmonary adenocarcinoma, lung infection

## Abstract

Jaagsiekte sheep retrovirus (JSRV) is the etiologic agent of ovine pulmonary adenocarcinoma (OPA), a neoplastic lung disease of sheep. OPA is an important economic and welfare issue for sheep farmers and a valuable naturally occurring animal model for human lung adenocarcinoma. Here, we used RNA sequencing to study the transcriptional response of ovine lung tissue to infection by JSRV. We identified 1,971 ovine genes differentially expressed in JSRV-infected lung compared to noninfected lung, including many genes with roles in carcinogenesis and immunomodulation. The differential expression of selected genes was confirmed using immunohistochemistry and reverse transcription-quantitative PCR. A key finding was the activation of anterior gradient 2, yes-associated protein 1, and amphiregulin in OPA tumor cells, indicating a role for this oncogenic pathway in OPA. In addition, there was differential expression of genes related to innate immunity, including genes encoding cytokines, chemokines, and complement system proteins. In contrast, there was little evidence for the upregulation of genes involved in T-cell immunity. Many genes related to macrophage function were also differentially expressed, reflecting the increased abundance of these cells in OPA-affected lung tissue. Comparison of the genes differentially regulated in OPA with the transcriptional changes occurring in human lung cancer revealed important similarities and differences between OPA and human lung adenocarcinoma. This study provides valuable new information on the pathogenesis of OPA and strengthens the use of this naturally occurring animal model for human lung adenocarcinoma.

**IMPORTANCE** Ovine pulmonary adenocarcinoma is a chronic respiratory disease of sheep caused by jaagsiekte sheep retrovirus (JSRV). OPA is a significant economic problem for sheep farmers in many countries and is a valuable animal model for some forms of human lung cancer. Here, we examined the changes in host gene expression that occur in the lung in response to JSRV infection. We identified a large number of genes with altered expression in infected lung, including factors with roles in cancer and immune system function. We also compared the data from OPA to previously published data from human lung adenocarcinoma and found a large degree of overlap in the genes that were dysregulated. The results of this study provide exciting new avenues for future studies of OPA and may have comparative relevance for understanding human lung cancer.

## Abstract (precis)

Ovine pulmonary adenocarcinoma is a chronic respiratory disease of sheep caused by jaagsiekte sheep retrovirus (JSRV). OPA is a significant economic problem for sheep farmers in many countries and is a valuable animal model for some forms of human lung cancer. Here, we examined the changes in host gene expression that occur in the lung in response to JSRV infection. We identified a large number of genes with altered expression in infected lung, including factors with roles in cancer and immune system function. We also compared the data from OPA to previously published data from human lung adenocarcinoma and found a large degree of overlap in the genes that were dysregulated. The results of this study provide exciting new avenues for future studies of OPA and may have comparative relevance for understanding human lung cancer.

## INTRODUCTION

Ovine pulmonary adenocarcinoma (OPA) is an infectious lung disease of sheep that is a significant economic problem and welfare concern for sheep producers in many countries (1, 2). The main clinical features of OPA are loss of condition and respiratory distress, and in many advanced cases, there is an accumulation of fluid in the lungs that drains from the sheep’s nostrils when its head is lowered (3). Lung function is often further compromised by the presence of bacterial or parasitic coinfection (1). By the time that clinical signs become apparent, tumor growth is typically extensive, and the disease is invariably fatal.

OPA is caused by jaagsiekte sheep retrovirus (JSRV), an exogenous betaretrovirus (4). Despite its etiological role in OPA, infected sheep produce only a limited adaptive immune response to JSRV antigens, and this has precluded the development of an effective preclinical serological diagnostic test or vaccine to control the spread of the disease (2). The mechanism underlying the poor immune responsiveness of sheep to JSRV is not completely understood. It appears likely that it is largely due to immunological tolerance elicited by the developmental expression of closely related endogenous JSRV (enJSRV) proteins in the ovine fetal thymus (5, 6). However, local immunomodulatory mechanisms are also proposed to contribute (7).

JSRV has a specific tropism for differentiated epithelial cells of the distal lung, and OPA tumor cells predominantly express markers of type II alveolar epithelial cells (AEC2s) (8–10). An unusual feature of JSRV is that the Env glycoprotein functions as a viral oncoprotein to drive neoplastic transformation *in vitro* (11, 12) and *in vivo* (13–16). JSRV Env expression activates a number of signaling pathways that control cellular proliferation, including phosphatidylinositol 3-kinase (PI3K)-Akt and mitogen-activated protein kinase (MAPK)–extracellular signal-regulated kinase 1/2 (ERK1/2) (17, 18). Several cellular factors that bind Env have been identified and are proposed to be involved in transformation (19–21), but further work is necessary to provide a complete model for Env-mediated tumorigenesis and to explain how this leads to the unique clinical presentation of OPA.

In addition to its veterinary importance, OPA represents a valuable animal model for some forms of human lung cancer due to similarities in histological appearance and the activation of common oncogenic signaling pathways (22–25). In its early stages, such as in subclinical natural disease and in experimentally infected lambs, OPA resembles a minimally invasive adenocarcinoma with a predominantly lepidic growth pattern (22, 24). In advanced natural disease, OPA is more closely similar to adenocarcinoma, with papillary or acinar predominant growth with or without mucinous features (22, 25). The similarity of OPA to human lung adenocarcinoma suggests that this naturally occurring sheep tumor could be valuable for understanding lung carcinogenesis, particularly at the early stages of disease, which are difficult to diagnose and study in humans.

In order to examine the pathogenesis of OPA, we determined changes in host gene expression in the lungs of lambs following experimental infection with JSRV. Many genes were identified to have altered expression, and we confirmed the upregulation of some of these using immunohistochemistry and reverse transcription-quantitative PCR (RT-qPCR). We also compared the differential gene expression of OPA-affected animals with previously published data on the two most common types of non-small-cell lung carcinoma (NSCLC) in humans: lung adenocarcinoma (LUAD) and lung squamous cell carcinoma (LUSC). Collectively, this study provides new information that greatly enhances our understanding of the host response to JSRV and provides a number of exciting new avenues for future work on OPA.

## RESULTS

### JSRV-induced gene expression in sheep lung tissue.

Whole-transcriptome profiling (RNA-Seq) was performed on lung tissue samples derived from JSRV-infected (*n* = 4) and mock-infected (*n* = 4) specific-pathogen-free (SPF) lambs. The samples used were obtained from a previous study (9), in which 6-day-old lambs were infected with JSRV by intratracheal injection and euthanized when signs of respiratory distress appeared (66 to 85 days postinoculation). Mock-infected lung tissue came from age- and sex-matched control lambs that had been inoculated with cell culture medium (9). OPA tumor lesions were observed in hematoxylin- and eosin-stained lung tissue sections from infected lambs and confirmed by immunohistochemistry (IHC) for the JSRV Env (SU) protein. Such lesions were not present in lung tissue from mock-infected lambs (9). Infected tissues contained many tumor foci distributed throughout the lung, which otherwise appeared histologically normal, a pattern typical of experimentally induced OPA (22). Samples for RNA-Seq were generated by pooling tissue from 7 distinct sites of each lung. In order to capture specimens that were representative of the whole tissue, the samples analyzed were not specifically enriched for tumor cells.

**FIG 1:**
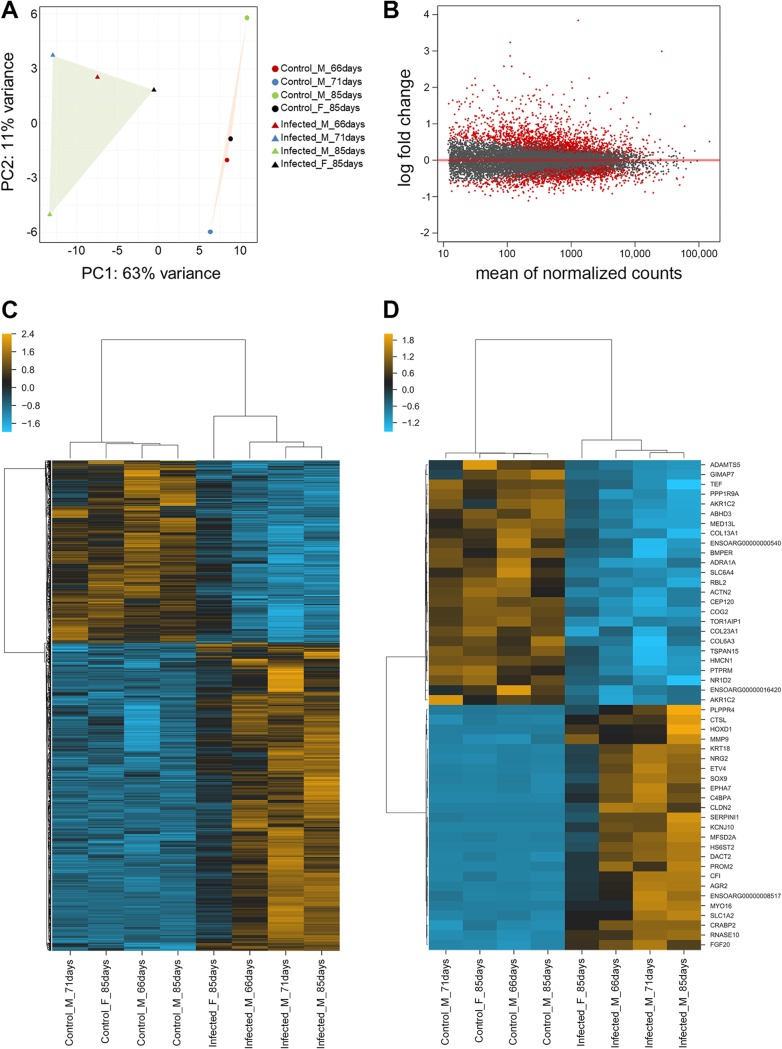
Host gene expression is modified in JSRV-infected lung tissue. (A) Principal-component analysis was performed using read counts from JSRV-infected and mock-infected (control) lambs for 15,149 genes mapping to the sheep genome. Each circle represents a mock-infected lamb, and each triangle indicates a JSRV-infected lamb. The values on each axis (principal component 1 [PC1] and PC2) represent the percentage of variance explained by each component. Principal component 1 separates the infected and mock-infected samples into two clusters with the highest variance of 63%. Within these clusters, there is greater variance in the JSRV-infected sheep than in the mock-infected sheep, and this likely corresponds to the proportion of tumor-affected tissue in the samples. For example, the lamb Infected_F_85days is the closest to the mock-infected cluster and had the lowest proportion of reads mapping to JSRV (Table 1), while the samples furthest from the mock-infected animals had the highest proportion of viral reads. (B) The gene expression data visualized as a two-dimensional scatter plot of the log_2_ ratio of expression values between infected and mock-infected tissue (i.e., the fold change) versus the mean expression across all samples. Each dot represents one gene, and the red color indicates the 1,971 genes identified to be differentially expressed between the infected group and the mock-infected controls using a false discovery rate (FDR) of <0.05. (C) Hierarchical clustering based on normalized gene counts of differentially expressed genes in samples derived from JSRV-treated and mock-infected lambs (1,237 were upregulated and 734 were downregulated). (D) Hierarchical clustering of the 25 most significantly upregulated and downregulated genes. F, female; M, male.

RNA-Seq generated over 60 million reads per sample, of which approximately 80% mapped uniquely onto the ovine and JSRV genomes (Table 1). A total of 15,149 sheep genes were identified, and the reads were quantified to identify those that were differentially expressed between mock-infected and JSRV-infected samples. Principal-component analysis of the normalized counts of sheep genes clearly separated the mock-infected and the JSRV-infected groups (Fig. 1A). JSRV infection produced a radical change in sheep gene expression, with 1,971 differentially expressed transcripts (1,237 upregulated and 734 downregulated) being identified between the two groups (Fig. 1B). We defined differentially expressed genes to be those showing up- or downregulation following JSRV infection with a false discovery rate (FDR) below 0.05, regardless of the observed fold change. The complete lists of mapped genes and differentially expressed genes are presented in Data Set S1 in the supplemental material. Hierarchical clustering of all differentially expressed genes is shown in Fig. 1C. Figure 1D summarizes the 25 most significantly upregulated and downregulated genes.

**TABLE 1:** Read mapping statistics

Lamb^*a*^	Total no. of reads	Total no. of reads quality trimmed	% of reads quality trimmed	Total no. of uniquely mapped reads	% of reads uniquely mapped	No. of viral reads	% of viral reads
1, Infected_M_66days	64,879,262	63,766,561	98.28	51,720,272	79.72	109,952	0.17
2, Infected_F_85days	72,907,048	71,678,589	98.32	58,431,025	80.14	68,763	0.09
3, Infected_M_71days	84,205,226	82,667,841	98.17	67,009,475	79.58	342,032	0.41
4, Infected_M_85days	68,631,006	67,438,835	98.26	55,039,775	80.2	196,608	0.29
5, Control_M_66days	60,417,140	59,431,332	98.37	48,778,008	80.74	54^*b*^	0.00
6, Control_M_85days	69,875,636	68,705,449	98.33	55,995,176	80.14	62^*b*^	0.00
7, Control_M_71days	64,020,136	62,964,748	98.35	51,447,365	80.36	128^*b*^	0.00
8, Control_F_85days	72,061,316	70,830,855	98.29	58,063,922	80.58	49^*b*^	0.00

a The lamb identifiers indicate the lamb number, infection status_sex (male [M] or female [F])_number of days postinoculation when culled.

b Reads mapping to the JSRV genome represented 0.09 to 0.41% of the total reads in the four infected lambs. A small number of reads mapping to JSRV (49 to 128 per sample) were also detected in the tissue from mock-infected control lambs. All of these reads mapped to regions of very high similarity between JSRV and enJSRV (data not shown) and so can be attributed to the transcription of endogenous viruses in the samples studied.

Although the RNA-Seq analysis revealed clear differences in gene expression between the infected and control groups, one infected lamb (Infected_F_85days) showed an intermediate overall expression pattern (Fig. 1A and C). Of the four infected lambs, this animal had the lowest percentage of reads mapping to JSRV in the RNA-Seq data (Table 1) and therefore likely had the smallest proportion of tumor-affected tissue and the largest contribution from healthy uninfected tissue. Nevertheless, hierarchical clustering grouped this animal with the other infected lambs (Fig. 1C and D), and so it was included in subsequent analyses.

### Functional analysis of RNA-Seq data.

**FIG 2:**
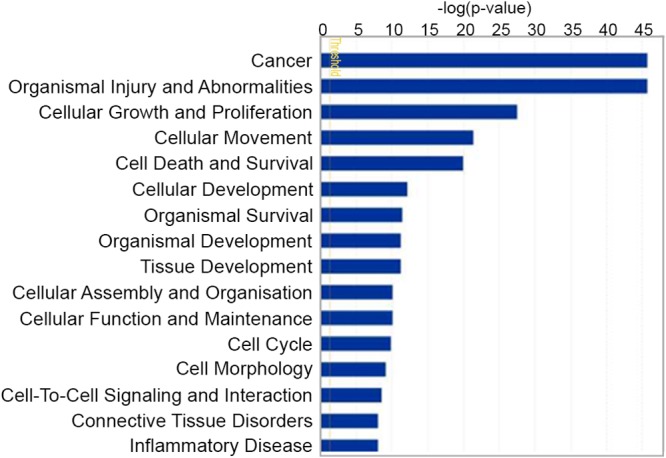
Main diseases and biofunctions associated with differentially expressed genes in JSRV-infected sheep lung. The figure shows the biofunction enrichment profiles identified by Ingenuity Pathway Analysis, plotted by relative statistical significance. Significance values were calculated based on a right-tailed Fisher’s exact test, and the −log(*P* value) is displayed on the horizontal axis of the bar chart. The taller the bar, the more significant the pathway effect.

**FIG 3:**
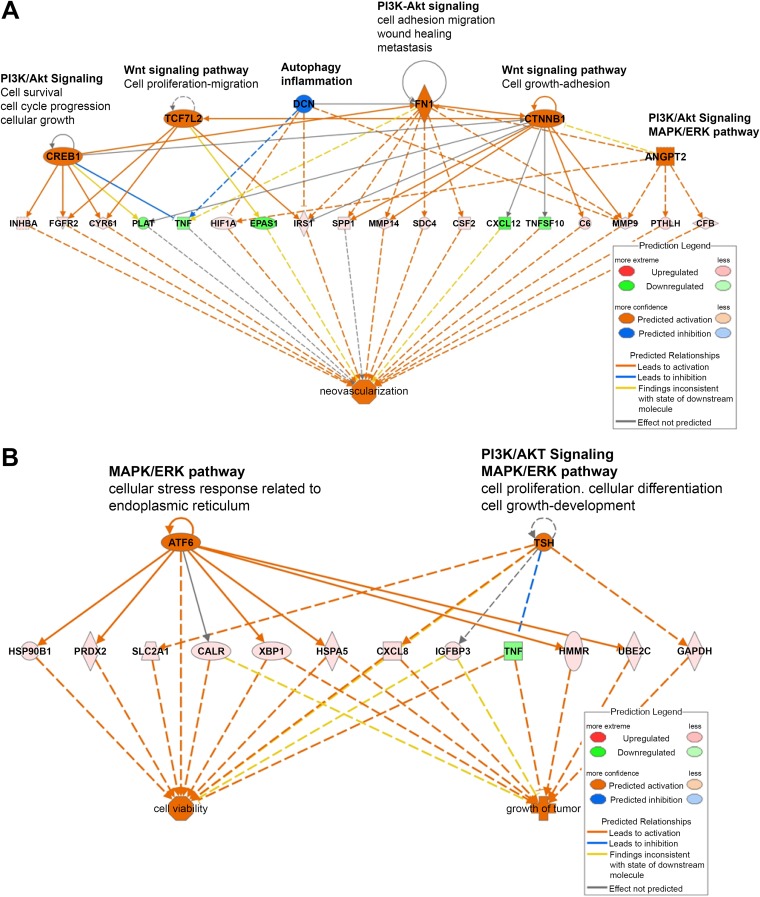
Mechanistic network analysis predicted by Ingenuity Pathway Analysis. The figure shows mechanistic network analysis by IPA of genes differentially expressed in experimentally induced OPA compared to mock-infected lung. (A) Neovascularization network. (B) Network for cell viability and tumor growth. The regulators are colored by their predicted activation state: activated (orange) or inhibited (blue). Darker colors indicate higher scores. The edges connecting the nodes are colored orange when leading to activation of the downstream node, blue when leading to its inhibition, and yellow if the findings underlying the relationship are inconsistent with the state of the downstream node. Pointed arrowheads indicate that the downstream node is expected to be activated if the upstream node connected to it is activated, while blunt arrowheads indicate that the downstream node is expected to be inhibited if the upstream node that connects to it is activated.

KEGG pathway enrichment analysis of the upregulated and downregulated genes was performed using the Database for Annotation, Visualization, and Integrated Discovery (DAVID) annotation software (https://david.ncifcrf.gov/ [26]) (Data Set S2). Upregulated pathways in the OPA-affected lung included genes involved in metabolic pathways, epithelial cell differentiation, cell cycle, and wound healing. Pathways involved in the immune response and inflammation were downregulated. Ingenuity Pathway Analysis (IPA) (27) was also used to functionally analyze the list of differentially expressed genes. The main diseases and biofunctions related to the differentially expressed genes are shown in Fig. 2. As with the DAVID analysis, cancer-related functions, such as cell proliferation and tissue development, were identified to be highly enriched, whereas those related to the immune response and inflammation were less strongly represented. These results revealed key signaling networks in cancer, including neovascularization, cell viability, and tumor growth, to be activated in JSRV-infected tissue compared to mock-infected tissue (Fig. 3). Based on these analyses, we used IHC to validate selected upregulated markers, focusing in particular on pathways of relevance to OPA pathogenesis, as discussed below.

### Cancer-related gene expression in OPA-affected lung tissue.

Many of the most significantly upregulated genes in OPA have previously been associated with important aspects of tumorigenesis in mice and humans. These include functions such as cell proliferation and differentiation (*CLDN2*, *CTSL*, *PROM2*, *AGR2*, *EPHA7*, *SOX9*, *KRT18*), angiogenesis (*MMP9*, *CCLA2*, *EPHB2*, *CXCL8*), metastasis (*CCLA2*, *EPHB2*, *AGR2*, *HS6ST2*, *MUC1*), and signal transduction, including epidermal growth factor (EGF) family members (*AREG*, *EREG*, *NRG2*) and Wnt signaling components (e.g., *WNT10B*, *PPARG*, *KRT18*, *LEF1*, *AXIN2*, *FZD3*, *FZD5*, *ROR1*, *CTNNB1*, and *CDH1*). In addition, factors previously identified to be specific markers of lung adenocarcinoma were upregulated, including *PROM2*, *CLDN3*, and *TJP3*, as were genes proposed to have potential diagnostic or prognostic value in human cancers, including *MMP9*, *AGR2*, *SULF1*, *NHSL1*, *LGR5*, *MUC1*, and *PIK3C2G*.

Although several factors related to angiogenesis were found to be upregulated using RNA-Seq, a previous study reported that vascular endothelial growth factor (VEGF) signaling was downregulated in OPA (28). The RNA-Seq data are consistent with the data from that study, finding reduced expression of VEGFD, VEGFA, and VEGFR2 (*KDR*) in JSRV-infected lambs, although only VEGFD passed our cutoff for statistical significance. The upregulation of MMP9 in OPA-affected lung tissue found by RNA-Seq is also in agreement with the findings of the previous study (28).

**FIG 4:**
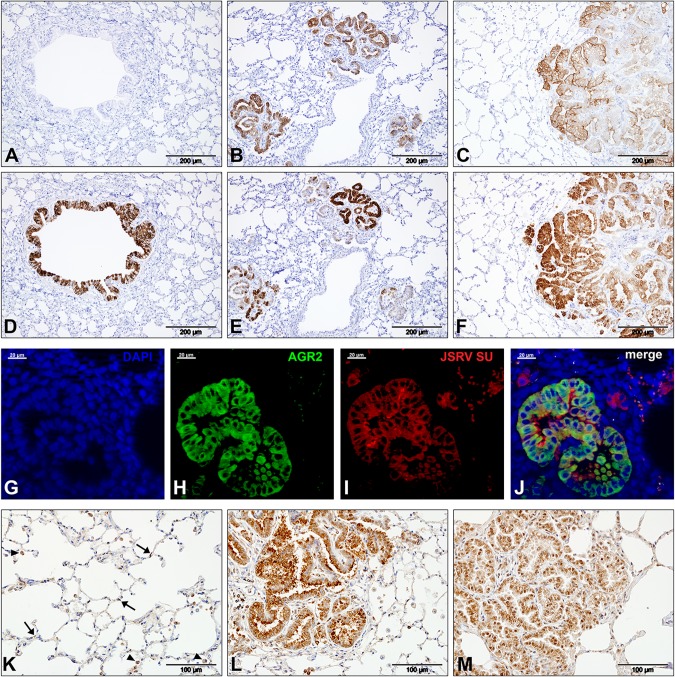
Immunohistochemical detection of AGR2 and AREG in JSRV-infected cells. (A to F) Immunohistochemical labeling of sheep lung with antibodies to JSRV SU (A, B, C) and AGR2 (D, E, F). (A and D) Serial adjacent sections of mock-infected sheep lung. (B and E) Serial sections of OPA lesions in the lung of a lamb experimentally infected with JSRV. (C and F) Serial sections of lung tissue from a natural case of OPA. Brown pigment indicates positive labeling. (G, H, I, J) Immunofluorescence labeling of lung tissue of an experimentally infected lamb. (H) AGR2 (green); (I) JSRV (red). (G) Staining with DAPI (blue), used to visualize nuclei; (J) the merged image. (K, L, and M) IHC labeling with an antibody to AREG. (K) Mock-infected lung shows labeling of some epithelial cells (arrows) and macrophages (arrowheads). (L) JSRV experimentally infected lamb; (M) naturally OPA-affected sheep lung showing the labeling of OPA lesions.

The upregulation of anterior gradient 2 (AGR2) was of particular interest, as this protein has been shown to promote oncogenesis in adenocarcinomas of several tissues, including the lung (29). To analyze the expression of AGR2 in OPA, IHC was performed on lung tissue sections from lambs with natural and experimental OPA and mock-infected lambs. The cytoplasm of bronchiolar epithelial cells from the mock-infected lungs labeled strongly for AGR2, but there was no labeling of cells in the alveolar compartment (Fig. 4A and D). In contrast, in the JSRV-infected lung, there were many regions of strong AGR2 labeling, and these correlated closely with areas of JSRV Env labeling in serial adjacent sections (Fig. 4B, C, E, and F). This was evident even on relatively small tumor foci, suggesting that AGR2 is activated early following JSRV infection. Immunofluorescent labeling confirmed the coexpression of JSRV Env and AGR2 in infected lung tissue, although not every infected cell cluster labeled positively for AGR2 (Fig. 4G to J). AGR2 has been shown to stimulate the expression of the EGF receptor (EGFR) ligand amphiregulin (*AREG*) in adenocarcinoma cells (30). AREG was also upregulated in OPA, and as for AGR2, IHC demonstrated positive labeling for AREG in OPA tumor cells from both natural and experimentally induced disease (Fig. 4K to M).

**FIG 5:**
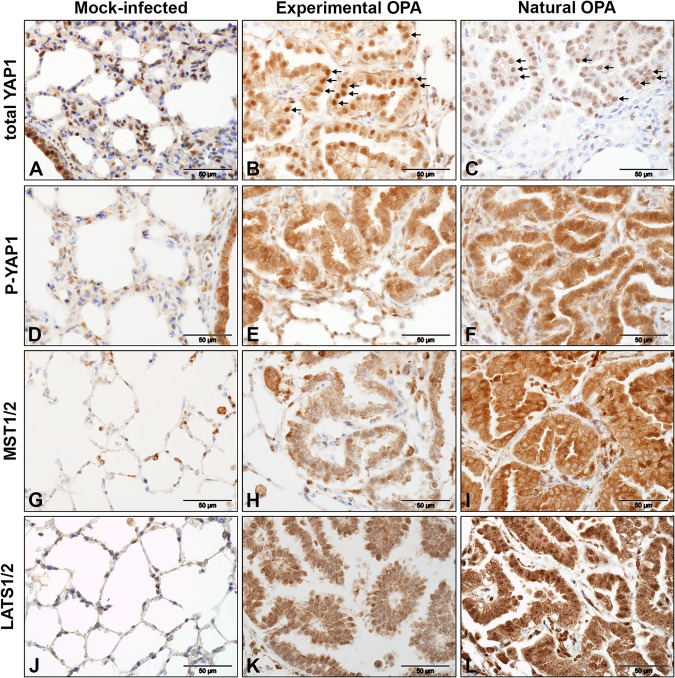
Immunohistochemical detection of Hippo pathway regulators in OPA-affected lung. IHC was performed on sections of ovine lung tissue with antibodies to proteins involved in Hippo pathway signaling. Left column, mock-infected sheep lung; middle column, experimentally infected lamb; right column, natural OPA. Brown pigment indicates positive labeling. (A to C) Total YAP1; (D to F) phosphorylated (inactive) YAP1 (P-YAP1); (G to I) MST1/2; (J to L) LATS1/2. Note that phosphorylated YAP1 expression is localized in the cytoplasm of tumor cells (E and F), whereas total YAP1 expression is shown in the nuclei and the cytoplasm of tumor cells (B and C). Black arrows in panels B and C indicate examples of YAP1 locating in the nucleus.

The activation of AREG by AGR2 is mediated by Yes-associated protein 1 (YAP1), a nuclear effector of the Hippo signaling pathway (30), which led us to ask whether Hippo signaling is involved in OPA. YAP1 was not found to be differentially expressed by RNA-Seq, but to examine this question further, IHC was performed on tissue sections of mock-infected and OPA-affected lung using antibodies to the phosphorylated form of YAP1 and to total YAP1 (Fig. 5). IHC detected both the phosphorylated and unphosphorylated forms of YAP1 in the bronchiolar epithelium and in the alveolar compartment of mock-infected lung (Fig. 5A and D). As expected, the phosphorylated (inactive) form was predominantly detected in the cytoplasm, whereas an antibody to total YAP1 (which detects the active and inactive forms) exhibited prominent nuclear labeling that was more intense than the labeling of the cytoplasm. Tumor cells in experimental OPA had strong nuclear labeling for total YAP1 (Fig. 5B), but this appeared to be less intense in natural OPA (Fig. 5C). IHC for regulators of the Hippo pathway, MST1/2 and LATS1/2, found that these proteins were readily detectable in AEC2s in healthy sheep lung (Fig. 5G and J), as previously reported in mouse lung (31). OPA tumor cells also labeled strongly for both markers (Fig. 5H, I, K, and L). Interestingly, the distribution of labeling for LATS1/2 differed between experimentally induced tumors and natural cases of OPA, being predominantly cytoplasmic in experimental disease and predominantly nuclear in natural disease. While a complete analysis of the activation state of the Hippo pathway is outside the scope of this study, these data indicate that the AGR2-YAP1-AREG axis is active in OPA and may contribute to oncogenesis in this disease.

### Immune response to JSRV infection and oncogenesis.

**FIG 6:**
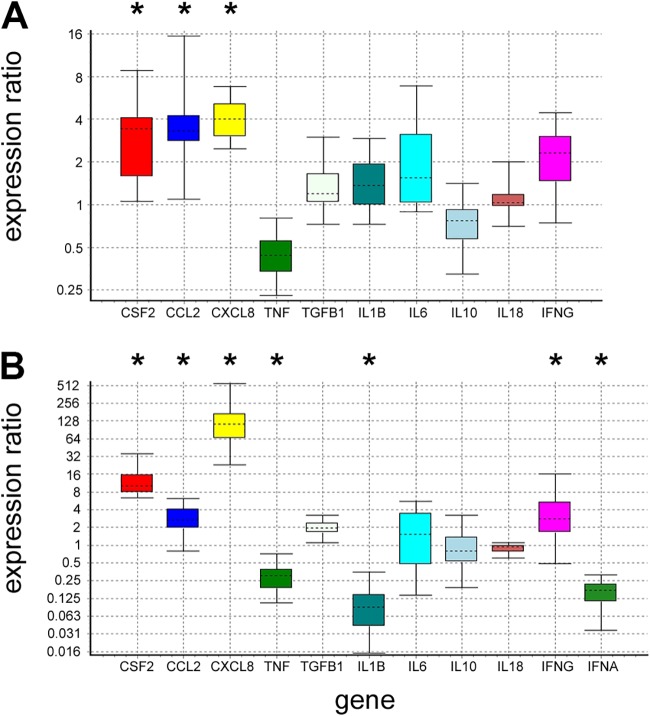
RT-qPCR detection of cytokine expression in OPA. The figure shows box-whisker plots of the relative expression of selected genes in OPA-affected lung tissue compared to healthy control tissue measured by RT-qPCR. (A) Expression in experimentally induced OPA relative to that in lung tissue from age- and sex-matched mock-infected lambs. (B) Expression in natural OPA relative to that in healthy adult sheep lung. Data analysis was performed using the software tool REST (72), which calculates the significance of the expression ratio based on the use of two reference genes and the amplification efficiency for each gene. Significant changes in expression are indicated by asterisks (standard error, <0.005 at *P* = 0.05). The dotted line within each box represents the median value, the boxed area encompasses the interquartile range, and the whiskers indicate the maximum and minimum data points. Note the difference in the scales on the *y* axes between the two charts. The data are consistent with the results of RNA-Seq analysis for these genes in experimentally infected lambs and controls, where *CSF2*, *CCL2*, *CXCL8*, and *IL-6* were upregulated, *TNF* was downregulated, and the expression of *IL1B*, *IL-10*, *IL-18*, and *IFNG* was not significantly changed. *TGFB1* and *IFNA* were not detected by RNA-Seq, although *TGFB3* was upregulated. (Note that *IFNA* was not examined in experimental OPA.).

The RNA-Seq analysis identified the altered expression of many genes related to immune responses, including various cytokines and chemokines (e.g., *CSF2*, *CCL2*, *CXCL6*, *CXCL8*, *CXCL14*, *IL1A*, *IL-6*, *TGFB3*, and *TNFSF18* were upregulated and *CSF1*, *CCL4*, *CCL18*, *CCL26*, *CXCL12*, *IL-15*, and *TNF* were downregulated), along with several members of the complement pathway (*C3*, *C5*, *C6*, *C7*, *MBL1* [*ENSOARG00000010165*], *CFI*, *CD46*, *PTX3*, *C4BPA*, and *C4BPB* were upregulated and *C1QA* and *C1QB* were downregulated), confirming a substantial host response to infection and tumor growth. Selected cytokines were also evaluated by RT-qPCR in experimentally induced and natural OPA, and this confirmed their differential expression (Fig. 6). Interestingly, the expression data do not support the strong induction of type I interferon responses in JSRV-infected lung. Although IFNA was not identified using RNA-Seq, RT-qPCR analysis found it to be downregulated in natural OPA (Fig. 6). Expression of IFNB1 was not significantly changed in JSRV-infected tissue. Furthermore, when considering a recently described panel of 90 highly conserved mammalian type 1 interferon-stimulated genes (32), only one (IRF7) was significantly upregulated in our data.

IFNG expression was not significantly upregulated in cases of experimental OPA, suggesting little or no activation of cellular Th1 responses in early disease. However, RT-qPCR found that IFNG was increased in tissue from cases of advanced natural OPA (Fig. 6B), consistent with a previous report of IFNG-positive macrophages in natural OPA (7). Markers of T lymphocytes were either not significantly changed (CD3 [gamma, delta, and epsilon chains] and CD4) or downregulated (CD8, alpha and beta chains). Interestingly, expression of *FOXP3* and *CD44*, both markers of regulatory T cells (Tregs), was significantly increased in OPA. Tregs negatively regulate T-cell immune responses through a number of mechanisms and have been shown to reduce the ability of CD8 T cells to produce tumor necrosis factor (TNF) (33). Consistent with this, *TNF* expression was downregulated in experimentally and naturally infected animals (Fig. 6).

**FIG 7:**
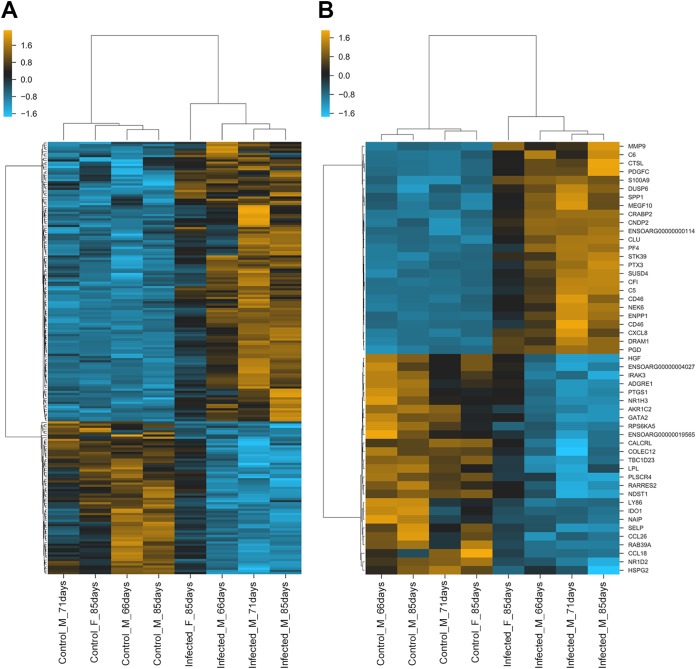
Expression of macrophage-related genes is altered in OPA. To identify macrophage-related functions in our data, a list of macrophage-related genes was compiled based on Gene Ontology (GO) terms (891 genes) and those present in macrophage coexpression clusters from the recently published sheep genome atlas (450 genes) (74) (see Data Set S3 in the supplemental material). Combining these two lists gave a total of 1,255 genes (86 were common to both sets), of which 1,076 were present in our RNA-Seq data and 198 were differentially expressed in JSRV-infected lung tissue. (A) Hierarchical clustering of 198 markers related to macrophage function that are differentially expressed in OPA. (B) Hierarchical clustering of the 25 most significantly upregulated and downregulated macrophage-related genes in OPA. Orange coloring indicates upregulation; blue indicates downregulation.

**FIG 8:**
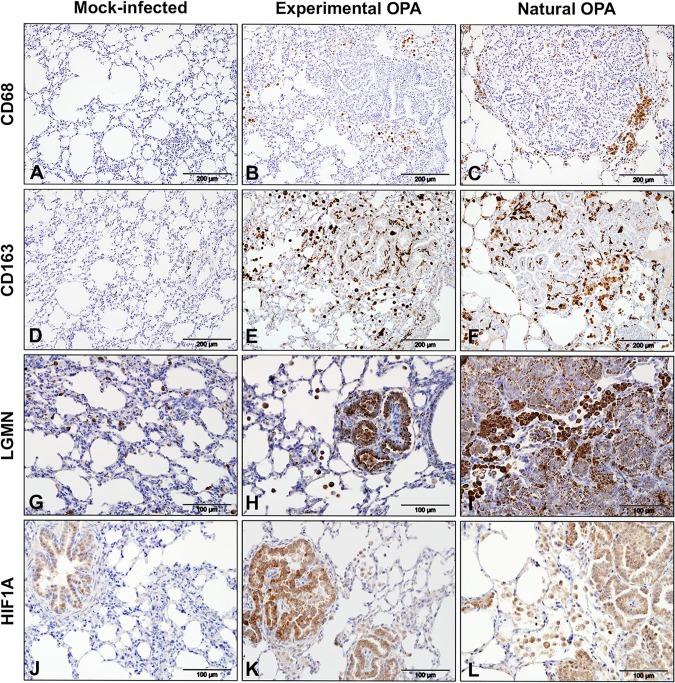
Immunohistochemical detection of macrophage markers in OPA-affected lung tissue. IHC was performed on sections of ovine lung tissue with antibodies to markers of macrophages. Left column, mock-infected lung; middle column, experimentally infected lung; right column, natural OPA-affected lung. Brown pigment indicates positive labeling. (A to C) Labeling with an antibody to CD68. (D to F) Serial sections of the tissues shown in panels A to C, respectively, labeled with an antibody to CD163. Note the difference in the distribution of labeling with the two antibodies. (G to I) Labeling with an antibody to LGMN; (J to L) labeling with an antibody to HIF1A. Note that antibodies to LGMN and HIF1A label some tumor cells, in addition to macrophages. In addition, LGMN exhibits a different pattern of labeling of tumor cells in experimental and natural OPA, where experimental cases have intense cytoplasmic labeling and natural cases have prominent labeling of the apical region.

In agreement with previous studies (7, 34), macrophages were observed in many sections of the experimental OPA cases studied here. In addition, approximately 10% (198 of 1,971) of the differentially expressed genes in our analysis have macrophage-related functions (Fig. 7; Data Set S3), confirming that macrophage-related gene expression is altered in JSRV-infected tissue. Upregulated genes included some previously associated with an inflammatory phenotype (e.g., *HIF1A*, *IL1A*, *CXCL8*, *CSF2*, *IRAK1*, *IRF7*) or an immunoregulatory phenotype (e.g., *CD163*, *CCL2*, *LGMN*, *MMP9*, *MMP14*), suggesting that a complex pattern of macrophage activation exists in OPA. Selected macrophage markers were also studied by IHC. Labeling for CD68, a commonly used marker of monocytes and tissue macrophages, identified cells located in normal alveolar lung tissue and along the margin of tumor foci (Fig. 8A to C). In comparison, an antibody to CD163, a marker for macrophages with an immunomodulatory phenotype (35), labeled many more cells, including cells that surrounded or infiltrated the tumor foci (Fig. 8D to F). Macrophages in or around OPA foci also labeled positively for legumain (LGMN), another marker associated with an immunoregulatory phenotype (Fig. 8G to I), and hypoxia-inducible factor 1-alpha (HIF1A), which is associated with an inflammatory macrophage phenotype (Fig. 8J to L). LGMN was also detected in alveolar macrophages in mock-infected tissue, while HIF1A labeling in mock-infected lung was limited to the bronchiolar epithelium. Interestingly, the antibodies to HIF1A and LGMN also labeled some tumor cells, whereas anti-CD163 and anti-CD68 did not. Consistent with the greater abundance of macrophages in OPA-affected lung, RNA-Seq identified the increased expression of several myeloid cell chemoattractants (e.g., *CCL2*, *CSF2*, *S100A8*, *S100A9*, and *CXCL8*) in JSRV-infected tissues.

### Comparison of gene expression of ovine pulmonary adenocarcinoma with human lung adenocarcinoma.

**FIG 9:**
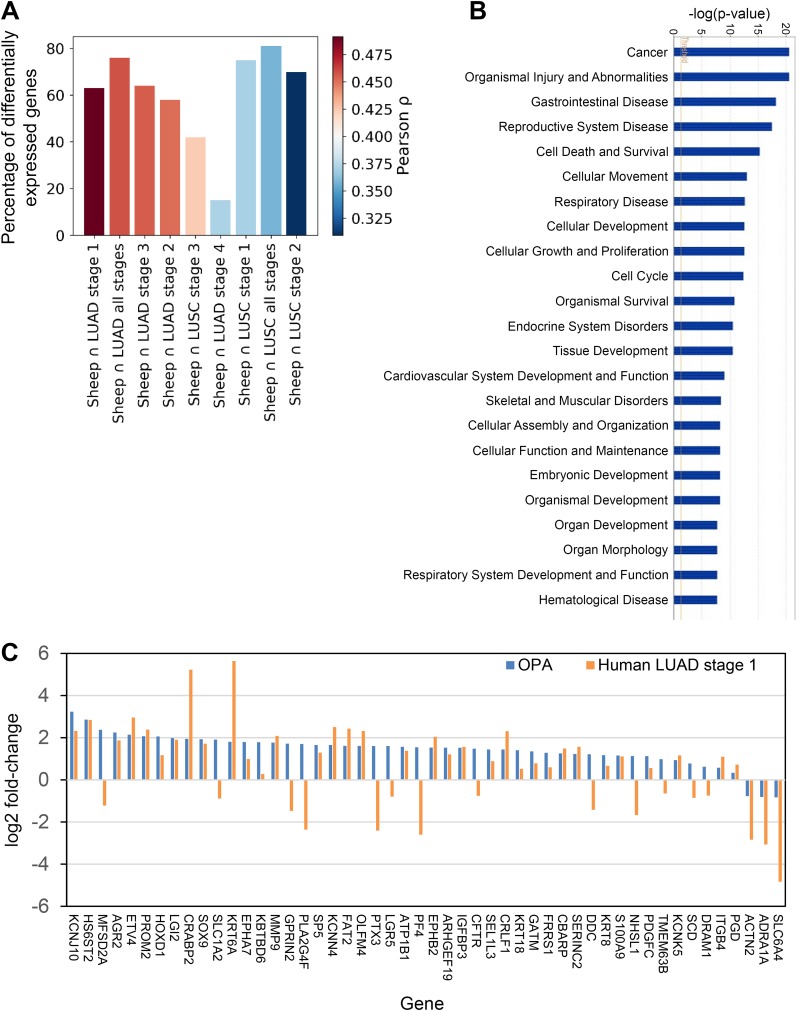
Comparison of gene expression in OPA and human lung cancer. Genes differentially expressed in experimentally induced OPA were compared with genes differentially expressed in human lung cancer, including clinical stages I to IV of LUAD and stages I to III of LUSC. (LUSC stage IV was not analyzed, as there were only two samples present in the data.) (A) Correlation plot comparing differentially expressed genes in OPA and in the different clinical stages of human LUAD and LUSC. The height of each bar shows the percentage of differentially expressed sheep genes that have differentially expressed orthologues in the human data set. The shading indicates the Pearson correlation coefficient between the log ratios of genes in common between the two lists. The data confirm a closer similarity of OPA to LUAD than to LUSC and to stage I LUAD in particular. (B) The differentially expressed orthologous genes in both sheep and human stage I LUAD were analyzed with Ingenuity Pathway Analysis software, and the figure shows the enriched diseases and biofunctions associated with those genes. (C) The plot shows the changes in gene expression in experimentally induced OPA and human stage I LUAD for the 50 most significantly changed sheep genes with deregulated human orthologues. Note that 37 of the 50 genes show a similar direction of change in expression, whereas 13 show divergent changes, highlighting the similarities and differences between the sheep and human diseases.

We next compared the RNA-Seq data from OPA with previously published expression data from the two most common types of NSCLC (LUAD and LUSC). Gene count data from RNA-Seq studies on tumors from patients with LUAD and LUSC were downloaded from The Cancer Genome Atlas (TCGA) database (portal.gdc.cancer.gov) (36). Data were selected from patients for whom matched normal and tumor data were available (57 LUAD and 49 LUSC patients; Data Set S4 shows their clinical annotation). The genes differentially expressed between tumor and normal samples were identified in each clinical disease stage (LUAD stages I to IV and LUSC stages I to III) (Data Set S5). To compare differentially expressed genes in sheep and human cancers, we considered only those sheep genes with one-to-one orthologues in humans (1,726 out of 1,971 [87%]). We then compared the lists of differentially expressed orthologous genes between OPA and the different stages of human LUAD and LUSC by calculating the Pearson correlation coefficient between the log ratios of genes in common between the two lists. The correlation plot of orthologous genes between OPA and the different stages of human LUAD and LUSC (Fig. 9A) revealed that the gene expression profile of OPA is more similar to that of LUAD than it is to that of LUSC and that the gene expression profile of OPA is more closely correlated with that of stage I LUAD than with that of later stages. The main diseases and biofunctions associated with the differentially expressed orthologous genes in OPA and stage I LUAD were identified by IPA (Fig. 9B). Data Set S6 lists the fold changes in expression of the genes differentially expressed in OPA with the changes in expression of their human orthologues in LUAD and LUSC and identifies those genes with similar or divergent changes in expression between the two species. Figure 9C summarizes the fold changes in expression of the 50 genes most significantly deregulated in OPA and the changes in expression of their human orthologues from stage I LUAD. Of these, 37 were consistent in their direction of change and 13 were discordant.

## DISCUSSION

The pathogenesis of OPA is intriguing. How do JSRV infection and expression of its oncogenic Env protein elicit the striking clinical and pathological phenotype typical of this disease? As the mechanisms underlying this process remain largely undetermined, here we used a global RNA-Seq approach to gain insight into the gene pathways and networks that are altered during the early stages of OPA. We identified 1,971 differentially expressed sheep genes and validated several of these by IHC or RT-qPCR. Some changes revealed the altered cellular composition of infected and uninfected lung. For example, the increased number of macrophages and the presence of tumor cells derived from AEC2s in infected tissue are reflected in the upregulation of markers of these cell types. However, many of the genes and pathways that have altered expression in JSRV infection have not been previously studied in OPA but are associated with important aspects of OPA pathogenesis, including tumor pathways and local immune responses. Collectively, this study provides important new information on the host response to JSRV infection that will be valuable for studies of OPA directly and for exploitation of the sheep disease as a lung cancer model.

In this study, we utilized tissue from experimentally infected SPF lambs in order to avoid the potential confounding effects of additional respiratory infections that commonly occur in natural cases of OPA (1). For welfare reasons, experimentally infected lambs must be culled when respiratory signs first appear, and therefore, the tissues studied represent those from an early stage of disease; in comparison, tissues from natural cases of OPA were from an advanced stage. We used sections of total lung tissue because we were interested in analyzing the gene expression of the whole infected tissue. A consequence of this approach is that the experimental OPA samples contained a greater proportion of histologically normal lung tissue than tumor tissue. We anticipated that this excess of healthy tissue would reduce the sensitivity of our study to detect differentially expressed genes, in particular, downregulated genes (37, 38). This would appear to be the case, as only four genes were found to have downregulated expression by more than 2-fold in OPA. Nevertheless, while this approach is expected to underestimate the fold changes of differentially expressed genes, it should still provide the correct order of significance. Future work will focus on analysis of gene expression in specific cell types, such as tumor cells and myeloid cells, which should increase the sensitivity of detection of differentially expressed genes in those cell subsets.

### Oncogenic signaling pathways in OPA.

The mechanisms involved in JSRV Env-mediated tumorigenesis are not completely understood, and multiple cellular pathways appear to be activated (15, 17, 18). Several studies have shown that Env expression leads to activation of the PI3K/Akt and MAPK/ERK1/2 signaling pathways (13, 18, 39, 40), while additional pathways, including EGFR and Wnt signaling, may also play a role (15, 19, 41, 42). The RNA-Seq data provided evidence supporting a variety of changes in gene expression related to carcinogenesis, including increased expression of several ligands of EGFR and other ERBB family receptors and upregulation of a variety of ligands and receptors related to Wnt signaling in JSRV-infected lung tissue.

Interestingly, the major components of the PI3K/Akt and MAPK/ERK1/2 signaling pathways were not significantly upregulated. Indeed, DAVID pathway analysis reported that both of these pathways were downregulated in OPA. There are several possible explanations for this apparent discrepancy. For example, activation of the MAPK and PI3K/Akt pathways is mediated by protein phosphorylation cascades, which may not correspond directly to changes in the transcription of those genes. In addition, while previous studies have shown roles for Akt and ERK signaling in tumor cells or transfected cell lines, the RNA-Seq analysis presented here reflects global transcriptional changes within the whole tissue, which comprises multiple cell types. Most importantly, the MAPK and PI3K/Akt pathways are involved in many cellular processes, in addition to oncogenesis, including immune responses (43, 44), and the list of genes that DAVID and similar tools use for identifying members of a pathway is very broad. For example, several of the genes identified by DAVID to be involved in the PI3K/Akt and MAPK/ERK1/2 pathways are involved in immune responses (e.g., *TLR2*, *CSF1*, *COL6A3*, *NFKB1*, and *TNF*), which is consistent with the other changes in the immune and inflammatory responses observed in our study.

An important novel finding in our study was the activation of AGR2 in OPA tumor cells (Fig. 4). AGR2 is a protein disulfide isomerase localized in the endoplasmic reticulum in normal cells but is upregulated in a variety of human adenocarcinomas, where it may also be present in secreted and cell-surface-bound forms (45, 46). AGR2 is associated with a poor prognosis in several cancer types and appears to mediate its oncogenic effect through the regulation of other genes, including *TP53* (47) and *AREG* (30), and through extracellular functions, such as promoting angiogenesis and extracellular matrix remodeling (46, 48). In humans, AGR2 expression is significantly higher in LUAD than in LUSC (49). The activation of AREG by AGR2 has been shown to be dependent on the Hippo pathway effector protein YAP1 in human adenocarcinoma cell lines (30). Consistent with this, IHC analysis confirmed the presence of nuclear YAP1 in OPA tumor cells (Fig. 5), suggesting a possible role for Hippo signaling in OPA pathogenesis. While further work is required, the transcriptional upregulation of AGR2, EGFR ligands, and other oncogenic factors identified by RNA-Seq provides new opportunities for understanding oncogenic signaling in OPA. In turn, OPA provides a model for studying the function of these pathways in a naturally occurring tumor, particularly at the early stages of tumorigenesis.

### Local immune responses in OPA-affected lung tissue.

The absence of a significant adaptive immune response to JSRV in sheep is commonly attributed to enJSRV expression in the fetal thymus during immune development, which is proposed to lead to immune tolerance through the deletion of T lymphocytes that recognize the closely related JSRV proteins (5). The results of the RNA-Seq analysis suggest that JSRV infection induces substantial immunological changes in lung tissue, including altered expression of numerous cytokines, chemokines, and complement factors, together with an increase in macrophages associated with tumor foci. These changes suggest that local immune-modulatory mechanisms active within the OPA-affected lung might also suppress the immune response to JSRV.

The activation of complement factors and complement regulatory factors in OPA is consistent with the findings of a previous microarray analysis of gene expression in a mouse model of JSRV Env-mediated transformation, which also found evidence of complement upregulation (50). Complement has previously been shown to inhibit infection with a number of viruses, including retroviruses (51), and studies on human and murine cancers have established roles for complement in modulating tumor growth (52–55). Therefore, complement might also play an immunomodulatory role in OPA tumors.

Macrophages exhibit significant functional plasticity (56, 57), and gene expression studies suggest the existence of numerous subpopulations of macrophages, including some with important roles in cancer (56–58). In addition, studies of mouse and human cancers have shown that macrophages and other myeloid cells in the tumor microenvironment are essential for tumor survival and metastasis (58, 59). Tumor-associated macrophages are also abundant in OPA (7), but the phenotype of these cells and their functional significance are unclear. In experimentally induced OPA, we identified changes in the expression of 198 genes related to macrophage function, including genes previously associated with an inflammatory or immunoregulatory phenotype. Interestingly, the expression of macrophage markers in OPA appears to vary depending on their location within the affected tissue, with cells on the periphery of tumor foci being CD68 positive and cells within the tumor being CD163 positive and CD68 negative (Fig. 8). CD163 is regarded as a reliable marker for immunomodulatory macrophages that are associated with a poor prognosis in human tumors (60, 61). The positive CD163 labeling of OPA-associated macrophages therefore suggests that these cells might also promote tumor growth in OPA.

In contrast, the transcriptome analysis provided little evidence to support a strong adaptive immune response in OPA. For example, there was no activation of T-cell markers, other than a modest but statistically significant increase in markers of Tregs (FOXP3 and CD44; although note that CD44 is also a marker for macrophages and AEC2s). In addition, there was no evidence of substantial activation of type 1 interferon responses in OPA, whereas IFNG expression was not significantly changed in JSRV-infected lambs but was upregulated in natural cases studied by RT-qPCR (Fig. 6). This is possibly due to the presence of concurrent bacterial infections in the natural cases but demonstrates an important difference between early experimental OPA and advanced natural cases.

Collectively, the gene expression data reported here suggest the presence of an immunomodulatory environment within the OPA lung which has the potential to suppress the immune response to JSRV and tumor cells and to actively promote tumor growth. A more detailed quantitative analysis of the role of tumor-associated myeloid cells, complement, and Tregs in OPA is necessary to determine their contribution to tumor growth and the development of clinical disease. This could reveal insights into the relevance of the tumor microenvironment to the apparent immune tolerance of sheep to JSRV and may inform the design of vaccine strategies for controlling OPA.

### Comparison of the transcriptomes of OPA and human LUAD.

As OPA is frequently cited to be an animal model for human lung adenocarcinoma, we compared the transcriptome data from OPA with published data on human lung tumors in an attempt to identify the similarities and differences between the diseases in the two species. The results indicate a closer similarity of the transcriptome of OPA with the transcriptome of human LUAD than with that of LUSC and, in particular, with the transcriptome of early disease (stages I and II) than with that of more advanced disease (stages III and IV) (Fig. 9A and C; see also Data Set S6 in the supplemental material). This is consistent with the histological appearance of OPA, which resembles a minimally invasive adenocarcinoma in its early stages (2, 22, 24, 25). It would be interesting in future studies to compare gene expression in the early stage of disease studied here with that in more advanced OPA.

Many genes showed a common pattern of differential expression in LUAD, LUSC, and OPA, but there were also many that did not. Some of these reflect differences between LUAD and LUSC and highlight the diversity of gene expression in the different tumor types. For example, 142 genes were upregulated in both OPA and stage I LUAD but downregulated or not significantly altered in stage I LUSC. Notably, this included *AGR2* and the gene for the Wnt pathway effector protein beta-catenin. Similarly, 86 genes were upregulated in OPA and stage I LUSC but not upregulated in stage I LUAD. In addition, there were 134 genes upregulated in OPA that were downregulated in both LUAD and LUSC. These included *SFTPC* and *LAMP3*, which are known markers of AEC2s in sheep and humans (62). LAMP3 has previously been detected in OPA but is rarely expressed in human tumors, except in bronchioloalveolar adenocarcinoma (lepidic-prominent adenocarcinoma *in situ*) (62), which was not represented in the LUAD cases in the TGCA data studied. Interestingly, the upregulation of complement factors observed in OPA was not evident in either LUAD or LUSC, and several other key immune response-related genes, such as *TLR10*, *CCL4*, *CCL26*, *CD8A*, *CD8B*, *TNF*, and *IDO1*, were specifically downregulated in OPA but not in human LUAD, suggesting that the innate immune responses in the sheep and human diseases may differ in important ways. Collectively, this analysis provides support for the similarity of experimental OPA to early stage LUAD but highlights that there remain many differences between the sheep and human diseases.

In summary, the findings from this first large-scale analysis of host gene expression in OPA significantly increase our understanding of the disease pathogenesis at a transcriptional level and will inform future research directed at improving OPA disease control. Moreover, the interspecies comparative data between sheep and humans provide additional support for the use of OPA as a model for early stages of LUAD, particularly noninvasive forms. Finally, a deeper understanding of the pathological changes of early tumors could help to identify novel biomarkers for the early detection of cancer lesions in both species.

## MATERIALS AND METHODS

### Animals and tissues.

Tissues were available from a previous study in which four SPF lambs were inoculated through intratracheal injection of JSRV at 6 days of age (9). Four additional lambs received cell culture supernatant (mock-infected control). Each group contained one female lamb and three male lambs. All lambs were delivered by caesarean section and housed under SPF conditions to minimize the risk of acquiring additional respiratory infections. Once the clinical signs of respiratory disease were apparent in the JSRV-infected animals (66 days, 71 days, and 85 days [*n* = 2] postinoculation), the lambs were culled. Each time a JSRV-infected lamb was euthanized, a healthy animal from the mock-infected control group was culled to provide age- and sex-matched control tissues. Tissues were collected from 24 locations in each lung and stored in liquid nitrogen for RNA extraction and in 10% buffered formalin for IHC. To study tissues from animals with natural disease, lung tissue samples were taken from four farm-raised adult sheep in the advanced stages of clinical OPA and four clinically healthy adult sheep. Cases which had no gross appearance of bacterial or parasitic infection were selected. All protocols involving animal handling and the use of postmortem material were approved by the Animal Welfare and Ethical Review Body of the Moredun Research Institute in accordance with the U.K. Animals (Scientific Procedures) Act 1986.

### RNA extraction and sequencing.

For RNA-Seq, RNA was extracted from frozen tissue samples from seven distinct sites of the lungs of each animal and pooled in equal amounts. Total RNA from cryosectioned tissue was extracted using an RNeasy minikit (Qiagen) according to the manufacturer’s instructions. RNA concentration and purity were measured (ND-1000 NanoDrop spectrometer), and RNA integrity was confirmed (with an Agilent RNA 6000 Nano kit with an Agilent 2100 bioanalyzer). All samples had an RNA integrity number (RIN) greater than 8.0. Prior to sequencing, frozen lung tissue sections derived from the same lung sites were examined by histology, which confirmed the presence of OPA tumor lesions in the JSRV-infected animals and the absence of lesions in the mock-infected negative controls. Total RNA was processed (TruSeq RNA Library kit) to generate cDNA libraries according to the manufacturer’s instructions and subsequently sequenced with an Illumina HiSeq2000 instrument using 100-bp paired-end sequencing (Edinburgh Genomics, University of Edinburgh, Edinburgh, UK).

### Processing of next-generation sequencing data and differential expression analysis.

Raw sequencing reads were processed to remove adaptors and poor-quality sequences (Q25 and below) using the Cutadapt (v1.10) program (63). Nonredundant reads were then mapped to the sheep (Oar_v3.1, Ensembl FTP release 74) and JSRV (GenBank accession number AF105220.1) genomes using HISAT2-2.0.4 software (64). The quantification of gene expression was calculated using HTSeq counts (65). Transcripts with fewer than 100 total reads across the eight samples were excluded. The sheep annotation (GTF) was obtained from Ensembl (Oar_v3.1.79) software. The virus and sheep gene counts were imported into the edgeR package (66), and the counts were normalized using a trimmed mean of M values (67) and fitted to a negative binomial generalized log-linear model to calculate the dispersion factor for each gene (68). Differentially expressed genes were then identified by applying an FDR cutoff of 0.05 (69). Principal-component analysis of normalized counts was performed using only sheep gene expression (i.e., counts from viral genes were removed) in order to see the variation between the two groups.

### Gene function annotation and pathway analysis.

Identification of enriched KEGG pathways in the upregulated and downregulated gene lists was performed with DAVID (v6.8) (26). Pathway analysis was performed using Ingenuity Pathway Analysis (IPA; v01-04) (27) to infer the functional roles and relationships of the differentially expressed genes based on the log_2_ fold change value of each gene.

### Comparative analysis with human data sets.

To compare significantly expressed genes in JSRV-infected lung with data from human NSCLC, HTSeq counts from RNA-Seq data for LUAD and LUSC were obtained from the GDC portal (portal.gdc.cancer.gov). Data for 57 LUAD patients and 49 LUSC patients for which corresponding normal and tumor data were available were selected. Differentially expressed genes in human tumors were identified using the edgeR pipeline, as described above for the sheep data. The list of differentially expressed genes in human data was filtered for only those that have an orthologous gene in sheep (identified using the Ensembl BioMart data mining tool) and were differentially expressed in sheep. The comparison of the expression of these short-listed genes was made using the correlation between the expression profiles (i.e., fold change) in humans and in sheep. The gene lists obtained are presented in Data Set S5 in the supplemental material.

### RT-qPCR.

RT-qPCR was performed according to MIQE recommendations (70). Primers and probes for reference and target genes are summarized in Table 2. RT-qPCR was performed using an ABI 7000 sequence detection system in 96-well plates (Applied Biosystems) with either TaqMan one-step RT-PCR reagents (Applied Biosystems) (for JSRV and CCL2) or a Power SYBR green RNA-to-CT 1-step kit (Applied Biosystems) (for all other target genes). Each sample (5 sites per animal) was tested in duplicate using 100 ng of RNA in a 20-μl final reaction volume. All experiments with SYBR green included a melting curve analysis to confirm the specificity of the amplicons (95°C for 15 s, 60°C for 20 s, and 95°C for 15 s). Standard curves constructed from 10-fold serial dilutions of positive-control RNA were used to determine the efficiency and replicate quality (*R*^2^) of each primer set. In addition, the level of gene expression in experimental samples was ensured to lie within the limits of the standard curve. For comparison of RNA transcription levels between samples, results from the RT-qPCR experiments were normalized to those for two reference genes, the genes for succinate dehydrogenase (SDHA) and β-actin (ACTB) (predetermined to be stable reference genes using geNORM software [71]). Statistical analysis for quantitative PCR was performed by group-wise comparison based on PCR efficiencies and the mean crossing point deviation between the sample and control group using the Relative Expression Software Tool (REST) (72).

**TABLE 2:** Primers and probes used in this study

Target^*a*^	Primer orientation or probe	Primer sequences (5′–3′)	Concn (nM)	Size (bp)	GenBank accession no. (reference)
ACTB	F	CTGAGCGCAAGTACTCCGTGT	300	125	NM_001009784 (75)
	R	GCATTTGCGGTGGACGAT	300		
SDHA	F	CATCCACTACATGACGGAGCA	200	90	AY970969 (75)
	R	ATCTTGCCATCTTCAGTTCTGCTA	200		
IFN-α	F	GCACTGGATCAGCAGCTCACTG	200	188	AY802984 (76)
	R	CTCAAGACTTCTGCTCTGACAACCT	200		
IFN-γ	F	TTCTTGAACGGCAGCTCTGAG	300	127	X52640 (77)
	R	TGGCGACAGGTCATTCATCA	300		
CSF2	F	GATGGATGAAACAGTAGAAGTCG	500	261	NM_001009805 (78)
	R	CAGCAGTCAAAGGGAATGAT	500		
TGF-β1	F	GAACTGCTGTGTTCGTCAGC	500	169	NM_001009400 (78)
	R	GGTTGTGCTGGTTGTACAGG	500		
TNF-α	F	GCCCTGGTACGAACCCATCTA	200	82	NM_001024860 (76)
	R	CGGCAGGTTGATCTCAGCAC	200		
IL-1β	F	CCTAACTGGTACATCAGCACTTCTCA	200	95	NM_001009465 (76)
	R	TCCATTCTGAAGTCAGTTATATCCTG	200		
IL-6	F	TCCAGAACGAGTTTGAGG	500	236	NM_001009392 (78)
	R	CATCCGAATAGCTCTCAG	500		
CXCL8	F	ACTGCGAAAATTCAGAAATCATTGTTA	500	53	S74436 (79)
	R	CTTCAAAAATGCCTGCACAACCTTC	500		
IL-10	F	AGCAAGGCGGTGGAGCAG	200	90	NM_001009327 (76)
	R	GATGAAGATGTCAAACTCACTCATGG	200		
IL-18	F	ACTGTTCAGATAATGCACCCCAG	200	100	NM_001009263 (76)
	R	TTCTTACACTGCACAGAGATGGTTAC	200		
CCL2	F	GCTGTGATTTTCAAGACCATCCT	900	72	DY503036 (80, 81)
	R	GGCGTCCTGGACCCATT	400		
	Probe	AAAGAGTTTTGTGCAGACCCCAACC	900		

a ACTB, β-actin; SDHA, succinate dehydrogenase subunit A; IFN-α and IFN-γ, interferon alpha and gamma, respectively; CSF2, colony-stimulating factor 2; TGF-β1, transforming growth factor β1; TNF-α, tumor necrosis factor alpha; IL-1β, interleukin-1β. Amplification conditions were 30 min at 45°C, 10 min at 95°C, and 40 cycles of 15 s at 95°C and 1 min at 60°C, except for CSF2 (30 min at 45°C, 10 min at 95°C, and 40 cycles of 20 s at 94°C, 30 s at 57°C, and 30 s at 72°C), CXCL8 (30 min at 45°C, 10 min at 95°C, and 40 cycles of 20 s at 94°C, 30 s at 55°C, and 30 s at 72°C), TGF-β1 (30 min at 45°C, 10 min at 95°C, and 40 cycles of 20 s at 94°C, 30 s at 55°C, and 30 s at 72°C), IL-6 (30 min at 45°C, 10 min at 95°C, and 40 cycles of 20 s at 94°C, 30 s at 52°C, and 30 s at 72°C), and CCL2 (30 min at 45°C, 10 min at 95°C, and 40 cycles of 20 s at 95°C, 30 s at 58°C, and 30 s at 60°C).

### Immunohistochemistry and immunofluorescence.

Tissue sections were routinely processed through graded alcohols and embedded in paraffin wax, and IHC was performed on sections (4 μm) mounted on charged glass microscope slides as previously described (9). The dilutions and sources of the primary antibodies used are described in Table 3. Isotype controls were used in semiserial tissue sections for each primary antibody. Additionally, the primary antibody was omitted to check for nonspecific labeling by the secondary antibody or the visualization system. Images for bright-field microscopy were examined using an Olympus BX51 microscope, and photographs were captured with an Olympus DP70 camera with analySIS software (Soft Imaging System GmbH, Munster, Germany). Immunofluorescence was performed as previously described (9) using primary antibodies to AGR2 (1:100; catalog number ab76473; Abcam), and JSRV Env (1:50) (73) with appropriate secondary antibodies conjugated with Alexa Fluor 488 (catalog number A11008; Molecular Probes) or Alexa Fluor 555 (catalog number A31622; Molecular Probes). Slides were mounted with medium containing 4′,6-diamidino-2-phenylindole (DAPI; Vectashield; Vector Laboratories). Images were analyzed using a Zeiss Axio Imager 2 fluorescence microscope with Apotome and AxioVision software.

**TABLE 3:** Summary of antibodies used for IHC

Target antigen	IHC dilution	Antibody type^*a*^	Reference or source, catalog no.
JSRV Env (SU)	1/200	Mouse MAb	73
AGR2	1/200	Rabbit MAb	Abcam, ab134167
AREG	1/200	Rabbit MAb	Abcam, ab224350
MST1/2	1/5000	Rabbit PAb	Abcam, ab87322
LATS1/2	1/2000	Rabbit PAb	Abcam, ab70565
Total YAP1	1/100	Rabbit MAb	Abcam, ab52771
Phosphorylated (s127) YAP1	1/300	Rabbit PAb	Abcam, ab76252
CD68	1/150	Mouse MAb	Dako, M0718
CD163	1/200	Mouse MAb	Bio-Rad, MCA1853
LGMN	1/400	Mouse MAb	Abcam, ab125286
HIF1A	1/50	Mouse MAb	Thermo Fisher, MA1-16511

a MAb, monoclonal antibody; PAb, polyclonal antibody.

### Data availability.

The raw RNA-Seq reads (fastq data) of each sample are present in the European Nucleotide Archive with the accession number PRJEB27638.

## Supplementary Material

Supplemental file 1

Supplemental file 2

Supplemental file 3

Supplemental file 4

Supplemental file 5

Supplemental file 6
